# Noninvasive ventilation reduces energy expenditure in amyotrophic lateral sclerosis

**DOI:** 10.1186/1471-2466-14-17

**Published:** 2014-02-07

**Authors:** Marjolaine Georges, Capucine Morélot-Panzini, Thomas Similowski, Jesus Gonzalez-Bermejo

**Affiliations:** 1 Sorbonne Universités, UPMC Univ Paris 06, UMR_S 1158 “Neurophysiologie Respiratoire Expérimentale et Clinique”, F-75005 Paris, France; 2 INSERM, UMR_S 1158 “Neurophysiologie Respiratoire Expérimentale et Clinique”, F-75005 Paris, France; 3 AP-HP, Groupe Hospitalier Pitié-Salpêtrière Charles Foix, Service de Pneumologie et Réanimation Médicale (Département “R3S”), F-75013 Paris, France; 4 Centre Hospitalier Universitaire de Dijon, Service de Pneumologie et Réanimation Respiratoire, Dijon, France

**Keywords:** Amyotrophic lateral sclerosis, Noninvasive ventilation, Energy expenditure, Diaphragm, Inspiratory neck muscles

## Abstract

**Background:**

Amyotrophic lateral sclerosis (ALS) leads to chronic respiratory failure. Diaphragmatic dysfunction, a major driver of dyspnea and mortality, is associated with a shift of the burden of ventilation to extradiaphragmatic inspiratory muscles, including neck muscles. Besides, energy expenditure is often abnormally high in ALS, and this is associated with a negative prognostic value. We hypothesized that noninvasive ventilation (NIV) would relieve inspiratory neck muscles and reduce resting energy expenditure (REE).

**Methods:**

Using indirect calorimetry, we measured REE during spontaneous breathing (REE_SB_) and NIV (REE_NIV_) in 16 ALS patients with diaphragmatic dysfunction, during the first 3 months of NIV. Measured values were compared with predicted REE (REE_pred_)(Harris-Benedict equation).

**Results:**

NIV abolished inspiratory neck muscle activity. Even though our patients were not hypermetabolic, on the contrary, with a REE_SB_ that was lower than REE_pred_ (average 11%), NIV did reduce energy expenditure. Indeed, median REE_NIV,_ in this population with a mean body mass index of 21.4 kg.m^-2^, was 1149 kcal/24 h [interquartile 970-1309], lower than REE_SB_ (1197 kcal/24 h, 1054-1402; mean difference 7%; p = 0.03, Wilcoxon). REE_SB_ and REE_NIV_ were correlated with forced vital capacity and maximal inspiratory pressure.

**Conclusions:**

NIV can reduce energy expenditure in ALS patients probably by alleviating the ventilatory burden imposed on inspiratory neck muscles to compensate diaphragm weakness. It remains to be elucidated whether or not, in which population, and to what extent, NIV can be beneficial in ALS through the corresponding reduction in energy expenditure.

## Background

Amyotrophic lateral sclerosis (ALS) is a degenerative disease that affects motor neurones in the cerebral cortex, brainstem and spinal cord, with ensuing atrophy of skeletal muscles. Respiratory failure develops when respiratory motor neurones are involved. ALS-related respiratory failure causes major suffering and is a leading cause of death [1]. Mechanical ventilation, most often administered non-invasively (NIV), is currently the only treatment for ALS-related respiratory failure. It prolongs survival and improves quality of life [2]. Diaphragm weakness is a major determinant of ALS-related respiratory failure [3,4].

A large proportion of ALS patients exhibit hypermetabolism [5-7], defined as an increase in resting energy expenditure (REE). Yet REE is a determinant of body weight and weight loss, that both have a documented negative prognostic impact in ALS [8-10].

Patients with ALS-related diaphragm weakness often exhibit strong phasic activity of inspiratory neck muscles —the so-called "respiratory pulse"— [3]. These muscles can be abnormally powerful at producing negative intrathoracic pressures for inspiration [11]. This can be interpreted as a compensatory mechanism to maintain ventilation: in ALS patients with diaphragm paralysis, vital capacity (VC) is directly correlated with the inspiratory pressure generating capability of inspiratory neck muscles [11]. In some cases, the phasic inspiratory activity of neck muscles extends during rapid-eye-movement (REM) sleep [4]. There therefore appears to be a shift of the inspiratory burden from the diaphragm to inspiratory neck muscles when the ALS degenerative process involves the phrenic motoneurones.

We hypothesized that extradiaphragmatic inspiratory muscles including inspiratory neck muscles contribute to "resting" energy expenditure in ALS patients with diaphragm weakness. To test this hypothesis we compared resting energy expenditure (REE) in ALS patients during spontaneous breathing and under NIV.

## Methods

### Patients

This was an exploratory "proof of concept" study conducted in a convenience sample of 16 patients with probable or certain ALS according to the revised El Escorial criteria [12] (Table 1). The study was conducted in the Paris (France) ALS reference center, in a 1600-bed tertiary university hospital. This study was conducted in accordance with the amended Declaration of Helsinki. The appropriate French regulatory and ethical authority (*Comité de Protection des Personnes Ile-de-France 6*, La Pitié-Salpêtrière, Paris, decision #102-12) approved the protocol, and written informed consent was obtained from all patients.

**Table 1:** Characteristics of ALS patients at initiation of ventilatory assistance and results of neurological and respiratory assessments

**Parameters**	**Median [1**^**st**^**-3**^**rd **^**quartiles]**
Anthropometric data	
Age (years)	68 [56.5-73]
Gender (male/female)	12/4
BMI (kg/m^2^)	21.4 [19.1-26.6]
Smoking (yes/no)	10/6
Neurological assessment	
ALS-FRS-R score	31 [26.5-35]
Norris bulbar score	35 [26.2-38]
Respiratory assessment	
Dyspnea score on MMRC scale	3 [1-3.5]
Inspiratory contraction of inspiratory neck muscles during quiet breathing in supine position (yes/no)	16/0
Inspiratory contraction of inspiratory neck muscles during indirect calorimetry in sitting position (yes/no)	11/5
PaCO_2_ (mmHg)	45 [42.5-48]
PaO_2_ (mmHg)	73 [66.5-77.5]
Bicarbonate (mmol/l)	28 [27-29.5]
Time spent with SpO_2_ < 90% (% of recording time)	30 [6.2-72]
FVC sitting (ml)	1990 [1140-2045]
FVC sitting (% predicted)	47 [35-54]
FVC supine (ml)	1650 [847-2182]
FVC supine (% predicted)	38.5 [30-58]
Pi_MAX_ (cmH_2_O)	31 [19.7-58.2]
Pi_MAX_ (% predicted)	37 [19.5-55.2]
SNIP (cmH_2_O)	32 [16.5-40.2]
SNIP (% predicted)	39 [23.2-42.7]

*Abbreviations*: *ALS-FRS-R* revised Amyotrophic Lateral Sclerosis - Functional Rating Scale, *BMI* Body Mass Index, *FVC* Forced Vital Capacity, *PaO*_*2*_ arterial oxygen tension, *PaCO*_*2*_ arterial carbon dioxide tension, *Pi*_*MAX*_ maximal inspiratory mouth pressure (measured from functional residual capacity; best of three maneuvers; data missing in 5 patients), *SNIP* Sniff Nasal Inspiratory Pressure (measured from functional residual capacity; best of ten maneuvers; data missing in 1 patients —in whom MIP was available—), *SpO*_*2*_ transcutaneous pulsed oxygen saturation.

Dyspnea was evaluated using the Modified Medical Research Council (MMRC) scale.

Grade 0: I only get breathless with strenuous exercise.

Grade 1: I get short of breath when hurrying on the level or walking up a slight hill.

Grade 2: I walk slower than people of the same age on the level because of breathlessness or have to stop for breath when walking at my own pace on the level.

Grade 3: I stop for breath after walking about 100 yards or after a few minutes on the level.

Grade 4: I am too breathless to leave the house or I am breathless when dressing.

#### Inclusion criteria

To be eligible for inclusion in this study, patients had to have been placed on NIV indication defined according to current criteria, [13] for at least 24 hours and up to 3 months (± 1 week). NIV had to be considered to be adequate, either immediately or after the first NIV adjustments [14]. Patients had to present signs of diaphragmatic dysfunction including respiratory pulse in the supine position.

#### Non-inclusion criteria

Patients with ALS in whom NIV had been started in an emergency context were not eligible. Patients with a disease other than ALS likely to alter nutritional status and metabolic status (renal failure, diabetes or thyroid disease, recent episode of acute respiratory failure, active infection, chronic pancreatitis, chronic alcoholism, corticosteroid therapy, known malignancy) could not be included in the study.

All in all, over the study period, 24 patients were eligible among 106 ALS patients seen at the center. Two of those refused to participate in the study, technical problems occurred in one case, and recordings were missed in 5 cases.

### Assessments

Neurological, respiratory and metabolic assessments were all performed on the same day.

#### Neurological assessment

The severity of the neurological deficit was evaluated by the revised ALS Functional Rating Scale (ALS-FRS-R), which rates the ability to perform activities of daily living from 0 (total inability) to 48 points (no limitation) and incorporates respiratory items (dyspnea, orthopnea, respiratory insufficiency). The bulbar section of the Norris scale was used to quantify bulbar impairment from 0 (no bulbar function) to 39 points (normal bulbar function). The date of onset of the symptoms and their initial level (bulbar or spinal), the date of confirmation of the diagnosis, and ongoing treatments (all patients received treatment with riluzole 50 mg, twice daily) were also recorded.

#### Respiratory assessment

Respiratory function was evaluated by arterial blood gases with spontaneous breathing in room air and by overnight pulse oximetry (SpO_2_) recording. Forced Vital Capacity (FVC) was measured in the supine and sitting positions with the EasyOne® ultrasound spirometer (NDD Medical Technologies, Andover, MA, USA). Inspiratory muscle strength was evaluated by measuring the maximal inspiratory pressure at the mouth (Pi_MAX_) and at the nostril ("sniff nasal inspiratory pressure", SNIP) using a Micro-RPM® digital manometer (Micro Medical, Chatham, Kent, UK).

Optimization of nocturnal NIV was verified by arterial blood gases performed in the morning, one hour after disconnecting the ventilator, nocturnal SpO_2_ recording on NIV and detection of leakages by the software integrated into the ventilator [14,15]. Of note, all the patients were equipped with home ventilators of the same make and model (Stellar®, Resmed, Bella Vista, Australia), with the spontaneous-timed pressure support mode (inspiratory and expiratory trigger).

#### Nutritional assessment

Nutritional assessment comprised measurement of height and weight for calculation of body mass index (BMI), by adopting a cut-off of 20 kg/m^2^ for malnutrition. Resting energy expenditure (REE) was measured by indirect calorimetry using a Quark RMR™ apparatus (Cosmed, Rome, Italy). Measurements were performed before 10 o’clock in the morning after fasting overnight and after resting for 20 minutes in a semi-sitting position in a quiet room heated to between 20°C and 24°C. Only values obtained at metabolic steady-state (less than 5% changes in V'O_2_, V'CO_2_ and RQ for at least 15 minutes) were taken into account.

**Figure 1:**
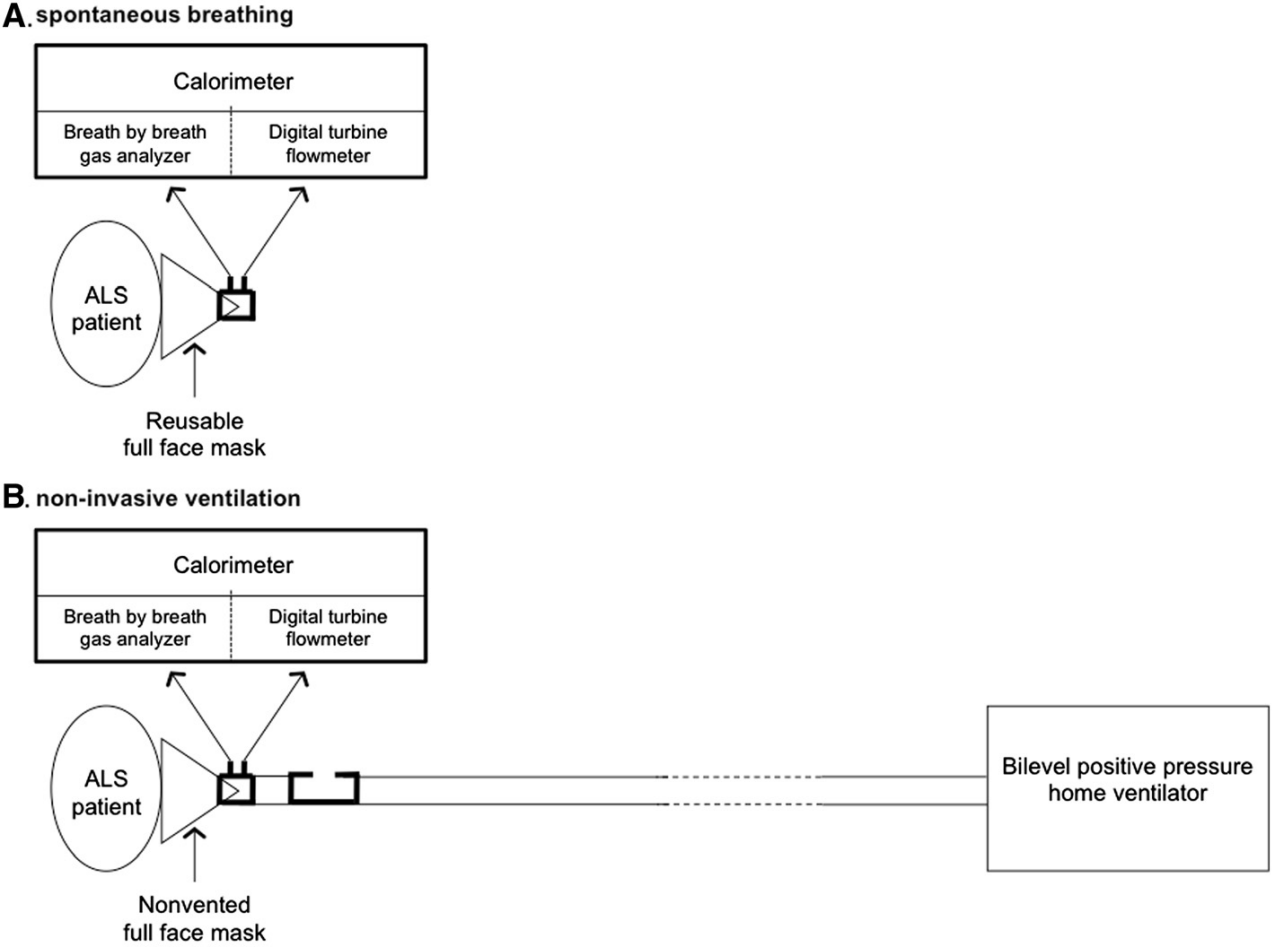
Schematic representation of measurement of energy expenditure during spontaneous breathing (A) and noninvasive ventilation (B).

During spontaneous breathing on room air (REE_SB_), oxygen consumption (V'O_2_) and CO_2_ production (V'CO_2_) were measured by a sensor fitted to the tip of a close-fitting oronasal mask. During NIV (REE_NIV_), inspired air is carried from the respirator to the mask via a single tube. Expired air is delivered into the chamber via a calibrated leak distal to the sensor measuring V’O_2_ and V’CO_2_ (Figure 1). This methodology was developed in several healthy subjects prior to the study to ensure that measurement of REE on spontaneous breathing by the canopy method was equivalent to the that obtained by the cycle-to-cycle gas analyzer and that the calibrated leak in the NIV circuit did not induce any reduction of REE.

Predicted REE (REE_pred_) was also calculated by the equation of Harris and Benedict [16]:

for males, REE = 66 + 1.38*weight(kg) + 5*height(cm)-6.8*age(years)

for females, REE = 655 + 9.7*weight(kg) + 1.8*height(cm)-4.7*age(years).

### Statistical analysis

Results were expressed as the median [1st quartile; 3rd quartile], and nonparametric tests were used for statistical analysis. REE measured on spontaneous breathing was compared to the predicted value by a Wilcoxon's test, which was also used to compare REE_NIV_ and REE_SB_. Correlations between metabolic status (REE_SB_, REE_NIV_, REE_pred_-REE_SB_, REE_SB_-REE_NIV_) and the various parameters likely to influence metabolic status were analyzed by Spearman’s rank correlation. The limit of significance was 5% (P < 0.05).

## Results

Measurements were performed after the first night on NIV in 10 cases, and at later time-points in 6 cases (after 32 to 96 nights). The anthropometric characteristics of the patients included in the study and the results of their respiratory and neurological assessments are summarized in Table 1. Thirty-one per cent (5/16) of patients presented an initially bulbar form of ALS. No patient has a gastrostomy. The first neurological symptoms had been present for an average of 26.5 [14; 39.2] months. All patients had received treatment with riluzole for an average of 13.2 [6.7; 19.5] months. At the time of the assessment, 37% (6/16) of patients had a BMI < 20 kg/m^2^ and 50% (8/16) of patients reported weight loss greater than 10% since onset of the disease. None of the patients had had a percutaneous endoscopic gastrostomy at the time of the procedure, but 6 of them (all among those who had lost weight) underwent this procedure in the following weeks.

REE_SB_ was significantly lower than REE_pred_ (1197.3 [1054.7; 1402.6] kcal/24 h vs 1389.5 [1193.9; 1622.6] kcal/24 h, p = 0.004). The mean REE_SB_ / REE_pred_ ratio was 90 [83; 97]%, with only one patient presenting a REE_SB_ / REE_pred_ ratio greater than 110% (111%).

Ventilation was increased on NIV, with correction of diurnal and nocturnal alveolar hypoventilation (Table 2). All patients reported complete or almost complete relief of dyspnea. Physical examination demonstrated that inspiratory neck muscle activity was abolished in every case.

**Table 2:** Evaluation of ventilatory variables on noninvasive ventilation

	**Spontaneous breathing in room air**	**Noninvasive ventilation median [1**^**st**^**-3**^**rd **^**quartiles]**	**p-value**
Vt (ml)	406.8 [289.5-486.8]	535.7 [450.8-578.4]	0.003
RF (/min)	17.6 [14.7-23.9]	15.4 [14.5-18.4]	0.02
Ventilation (l/min)	7.1 [6.5-7.9]	8.3 [7.5-9.9]	<0.001
PaCO2 (mmHg) (n = 12)*	47 [43-48]	41.5 [38-44]	0.002
PaO2 (mmHg) (n = 12)*	69 [65.7-75.2]	88.5 [70-97.5]	0.005
A-a gradient (mmHg) (n = 12)*	23.1 [16.6-27.6]	9.2 [3.9-29.3]	0.03
Vd (ml) (n = 12)*	117.7 [78.2-141.6]	178.9 [152.6-211.8]	0.01
Time spent with SpO_2_ < 90% (% of recording time)	30 [6.2-72]	1 [1-3]	<0.001

*Abbreviations*: *A-a* gradient for Alveolar-arterial gradient, *PaO*_*2*_ arterial oxygen tension, *PaCO*_*2*_ arterial carbon dioxide tension, *RF* Respiratory Frequency, *SpO*_*2*_ transcutaneous pulsed oxygen saturation, *V*t for tidal volume, *V*d dead space volume (calculated using Bohr formula, note that non invasive ventilation efficiently corrects PaCO2 in spite of an increased Vd -expected-).

**Figure 2:**
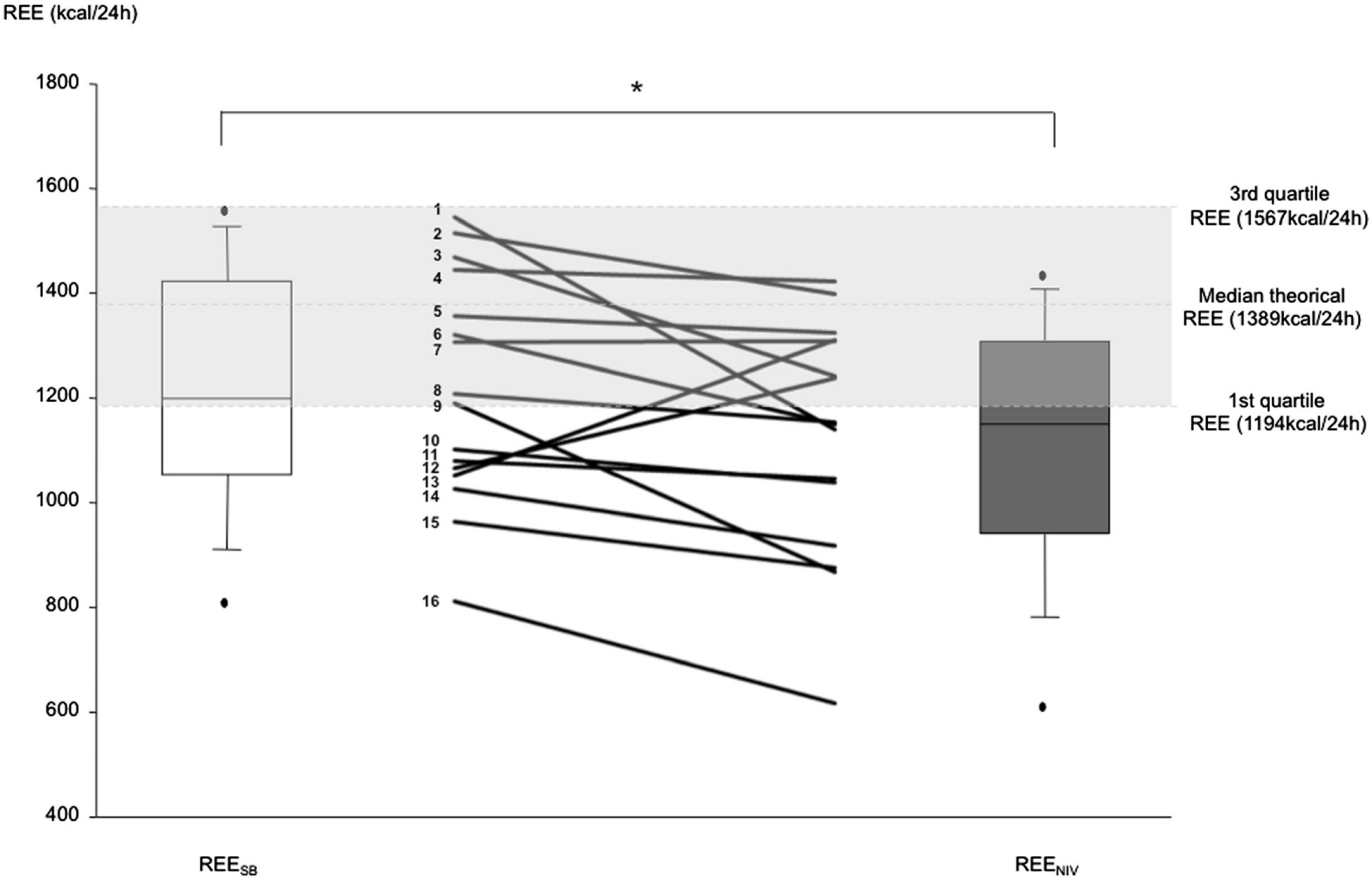
**Resting energy expenditure (REE) in the study population (box-and-whiskers) with indication of individual data (solid lines) during spontaneous breathing (REESB) and noninvasive ventilation (REE**_**NIV**_**).** The limits of the boxes correspond to the 25th and 75th percentiles of the distribution, with indication of the median as the black solid lines within the boxes. The whiskers represent the 10th and the 90th percentiles, respectively, and the black dots indicate the extreme values. The gray area corresponds to the interquartile space of REE predicted according to the equation of Harris and Benedict.

REE was significantly decreased on NIV (REE_NIV:_ 1149.2 [970.8; 1309.5] kcal/24 h, p = 0.03 compared to REE_SB_; REE_SB_ - REE_NIV:_ -78.1 [-186.2; -27.5] kcal/24 h, i.e. by -7 [-14; -2]% (Figure 2). There was no difference in the magnitude of this result between the 10 patients studied immediately after NIV initiation and the 6 patients studied later.

REE_SB_ and REE_NIV_ were significantly correlated with gender, the Norris bulbar score, sitting FVC and Pi_MAX_ (Table 3). There was no other correlation with any of the anthropometric (BMI, age), respiratory or neurological (severity, history of disease) variables. The REE_SB_ - REE_NIV_ difference did not correlate with any of the collected variables.

**Table 3:** Significant correlations between resting energy expenditure and gender, Norris bulbar score, forced vital capacity and maximal inspiratory mouth pressure

	**REE**_**SB**_		**REE**_**NIV**_		**REE**_**SB**_**-REE**_**NIV**_	
Gender	0.523* 0.037-0.809	0.038	0.714* 0.337-0.893	0.001	0.282 -0.248;0.683	0.289
Norris bulbar score	0.582* 0.099-0.843	0.023	0.677* 0.253-0.883	0.005	0.157-0.386;0.620	0.575
FVC sitting (ml)	0.577* 0.038-0.856	0.039	0.635* -0.022-0.821	0.019	0.079-0.494;0.604	0.798
Pi_MAX_ (cmH_2_O)	0.826* 0.359-0.962	0.006	0.707* 0.080-0.933	0.033	-0.044 -0.688;0.639	0.910

REE, resting energy expenditure; "sb", during spontaneous breathing; "niv", during non invasive ventilation.

FVC, Forced Vital Capacity; Pi_MAX_, maximal inspiratory mouth pressure.

## Discussion

This study shows that NIV can acutely decrease resting energy expenditure in ALS patients with ALS-related chronic respiratory insufficiency exhibiting signs of severe diaphragmatic dysfunction.

### Study limitations

For the purposes of this proof-of-concept study, we deliberately selected a small number of patients who were as homogeneous as possible in terms of the respiratory impact of ALS. Apart from NIV criteria, all patients exhibited obvious signs of diaphragmatic dysfunction including increased activity of inspiratory neck muscles, which may limit extrapolation of our results. Of note, the diagnosis of diaphragmatic dysfunction was based on physical examination and phrenic nerve stimulation was not performed to measure diaphragm strength and to distinguish lower from upper respiratory motor neurone diseases [3]. Silencing of inspiratory neck muscles by NIV was also not confirmed by electromyography (but only clinically). If anything, residual inspiratory neck muscle activity would have underestimated the effect of NIV on energy expenditure. Similarly, the patients were studied under a triggered NIV modality: controlled mechanical ventilation could have resulted in a larger reduction of REE. Measurement errors with indirect calorimetry are a major concern, particularly when comparing two conditions involving different setups. We took extensive precautions to control for technical issues that could have interfered with the measurements, and extensive preliminary measurements were performed in normal subjects to check that REE was not affected by the different setups. The difference between REE_SB_ and REE_NIV_ in our patients (median 7%) exceeded the coefficient of variation of the measurement reported by other authors under similar conditions 3.5% ± 2.7% -standard error- in stable mechanically ventilated patients, see [17] and was also greater than the variability observed in normal subjects during our in-house preliminary experiments.

### Comparison with available data

#### REE and mechanical ventilation: the energy cost of breathing

The contribution of respiratory muscles to REE or oxygen consumption at rest has been estimated in the literature to be between 1% [18,19] and 5-7% in normal individuals [20-23] and in quadriplegics [24]. These estimates were mostly based on back-extrapolations of the relationship between V'O2 and load-induced increases in the work of breathing [25]. This relationship is not linear and can underestimate the oxygen cost of unloaded breathing. To avoid this bias, the energy cost of breathing can be derived from comparisons between energy expenditure measured during spontaneous breathing and during mechanical ventilation in the same individuals [26,27]. This approach implies that respiratory muscles are actually passive during the measurements performed under mechanical ventilation, which is not always easy to ascertain. In patients with chronic obstructive pulmonary disease receiving NIV, Hugli et al. [28] estimated that respiratory V'O_2_ was an average of 1.6% of V'O_2_. This figure was underestimated because NIV mostly failed to abolish respiratory muscle electromyographic activity. In addition, the ventilation-induced reduction in V'O_2_ was not normally distributed (1.6 ± 6%) and some patients had respiratory V'O_2_ values of about 10% of V'O_2_[28]. In quadriplegic patients treated with phrenic stimulation, switching ventilatory assistance from controlled mechanical ventilation to diaphragm pacing increased REE by an average of 21% [29]. Because the patients in this study were overventilated, their respiratory-related energy expenditure was probably artefactually high. Hyperventilation-corrected values fell in the 10-15% range. To the best of our knowledge, the study with the most similar design to the present study compared energy expenditure during spontaneous breathing and mechanical ventilation in 9 tracheotomized patients with post-polio chronic respiratory failure [30]. In these patients, REE decreased from 1378 (958—1607) kcal/24 h during spontaneous breathing to 1086 (598—1579) kcal/24 h during pressure support ventilation (22%), and V'O_2_ decreased from 207 (141—235) ml/min) to 159 (92—230)(23%). The REE reduction observed during NIV in our ALS patients (median 7%) and it V'O_2_ counterpart are therefore in the upper range of available normal values for respiratory V'O_2_ and in the lower range of reported values in patients. In addition to the possible underestimation discussed above, it must be emphasized that our patients mostly depended on their inspiratory neck muscles to breathe, as their diaphragm did not participate in ventilation.

#### REE in ALS

In our patients, REE_SB_ was on average 10% lower than the value predicted by the Harris and Benedict equation. Of note, in the literature the prediction error for the Harris-Benedict equation has been reported to be as high as 18.6 + 14.9%, with limits of agreement showing that this equation could overestimate caloric expenditure by 591 kcal/d and underestimate requirements by 677 kcal/d [31,32]. The energy requirements in ALS is a complex issue see review in [33] and the details are beyond the scope of this discussion. Some studies have shown that one-half to two-thirds of ALS patients can be described as "hypermetabolic" [5-7] and remain so throughout the course of the disease [5]. In comparison with these studies, our patients had a slightly lower BMI, which, although fat-free mass was not measured, tends to suggest that they were somewhat malnourished. More importantly, the respiratory status of our patients was poorer than that reported in other studies, as our study appears to be the first to provide REE measurements during spontaneous breathing in ALS patients meeting the criteria for mechanical ventilation (Ellis and Rosenfeld measured REE in ALS patients requiring NIV, but it is unclear from their article whether the measurements were obtained during spontaneous breathing or under NIV) [32]. Our patients had a mean FVC of 42.7 ± 5.9% when REE_SB_ was determined, compared with a mean FVC close to 80% in the studies by Desport et al. [6,7]. In the longitudinal study by Bouteloup et al. [5], the 28 patients with sequential REE measurements had a mean VC of 67% at the time of the last measurement. In the longitudinal study by Kasarkis et al [34], performed before the NIV era, some patients were and remained hypermetabolic during the course of the disease, but the lowest observed FVC in this population was greater than 50%. It is therefore possible that the development of respiratory insufficiency in ALS has a masking effect on hypermetabolism, as suggested by Vaisman et al. [35]. This could occur via various mechanisms, including increasing malnutrition [33,35] due to the combination of ALS-related impairment of swallowing and/or eating-related dyspnea [36], as described in other types of severe chronic respiratory insufficiency [37,38]. Of note, energy expenditure in ventilated ALS patients has been found to be low [8,39], with results comparable to those observed in ventilator-dependent Duchenne patients [40], but it has also been found to be increased. Sherman et al. observed hypermetabolism in ventilated patients [31], and Ellis and Rosenfeld observed moderately increased REE values in patients requiring NIV [32]. In both cases, mean BMI values were about 25 kg.m^-2^, vs. 22 in our patients, which might explain the discordant results. In any case, the present study showed that REE decreased under NIV in patients who were not hypermetabolic but who tended to be hypometabolic (possibly because of some degree or malnutrition): the amount of energy expenditure spared by NIV would probably have been even greater in better nourished patients. Also worthy of notice, the effect of NIV on REE was quite heterogeneous in our patients (Figure 2). We did not evidence any particular explanation for this finding. The reduction in REE was similar in the 10 patients studied upon NIV initiation and in the 6 patients studied somewhat later, and the correction of hypoventilation was excellent and homogeneous in the study population (Table 2). In addition, the REE response to NIV did not correlate with any of the anthropometric, respiratory or neurological variables collected.

## Conclusions

There is a general consensus that NIV prolongs survival and improves quality of life in ALS patients, and particularly those without severe bulbar dysfunction. These positive outcomes are implicitly attributed to the correction of hypoventilation and nocturnal desaturations, and to the relief of dyspnea. In the light of the present observations, it might be hypothesized that NIV could also be beneficial, in certain cases, by reducing energy expenditure and therefore contributing to a better nutritional equilibrium —the prognostic value of which has been established—. Data from Lechtzin et al. [41] have suggested that starting NIV early in the course of respiratory involvement can be associated with improved survival (an average gain of one year in a population with a mean FVC of 74.3 ± 10.1% pred —absence of obvious respiratory insufficiency—, in comparison with a population with a mean FVC of 48.3 ± 11.3% pred). These results must be taken with caution because they have not been prospectively corroborated and could, at least in part, be due to a time bias effect. However, our observations point at a putative mechanism for a beneficial effect of early NIV, namely a reduced respiratory-related energy expenditure. Of note, the accelerated muscle wasting due to hypermetabolism is bound to involve respiratory muscles. A NIV-related reduction in energy expenditure could therefore theoretically contribute to slow respiratory decline, yet a recent retrospective study suggested, apparently for the first time, that NIV could indeed decrease the ALS-related decline of VC [42]. The correlation observed between REE and FVC and Pi_MAX_ in our patients (Table 3) tends to supports this hypothesis. Nevertheless, NIV may prove difficult to implement in asymptomatic patients. Proposing "early NIV" with the purpose of reducing energy expenditure would require prior convincing documentation of the corresponding clinical benefits. Further studies are therefore needed to elucidate what determines the response of REE to NIV and whether, in what particular population, and to what extent, early NIV can be beneficial in ALS through a reduction in respiratory-related energy expenditure. The role of ventilatory mode and triggering modalities is among the factors to be studied.

## Abbreviations

ALS: Amyotrophic lateral sclerosis; ALS-FRS-R revised: ALS functional rating scale; BMI: Body mass index; FCV: Forced vital capacity; INM: Inspiratory neck muscles; NIV: Noninvasive ventilation; PiMAX: Maximal inspiratory pressure; REE: Resting energy expenditure; REESB: REE measured during spontaneous breathing; REENIV: REE measured during noninvasive ventilation; REEpred: Predicted REE according to the Harris and Benedict equation; REM: Rapid eye movement sleep; SNIP: Sniff nasal inspiratory pressure; SpO2: Pulsed oximeter oxygen saturation; V’CO2: Carbon dioxide production; V’O2: Oxygen consumption.

## Competing interests

This study did not involve any competing interest, financial or otherwise, for any of the authors.

## Authors’ contributions

MG participated in the conception and design of the study, performed most of data collection and analysis, participated in data interpretation, drafted the submitted article and approved the final submission. CMP participated in the conception and design of the study, contributed to data analysis and data interpretation, revised the manuscript and approved the final submission. TS participated in the conception and design of the study, contributed to data analysis and data interpretation, revised the manuscript and approved the final submission. JGB participated in the conception and design of the study, contributed to data collection, data analysis and data interpretation, revised the manuscript and approved the final submission.

## Pre-publication history

The pre-publication history for this paper can be accessed here:

http://www.biomedcentral.com/1471-2466/14/17/prepub

## References

[B1] Gil J Funalot B Verschueren A Danel-Brunaud V Camu W Vandenberghe N Desnuelle C Guy N Camdessanche JP Cintas P Causes of death amongst French patients with amyotrophic lateral sclerosis: a prospective study Eur J Neurol 2008 15 1245 1251 10.1111/j.1468-1331.2008.02307.x 18973614

[B2] Bourke SC Tomlinson M Williams TL Bullock RE Shaw PJ Gibson GJ Effects of non-invasive ventilation on survival and quality of life in patients with amyotrophic lateral sclerosis: a randomised controlled trial Lancet Neurol 2006 5 140 147 10.1016/S1474-4422(05)70326-4 16426990

[B3] Similowski T Attali V Bensimon G Salachas F Mehiri S Arnulf I Lacomblez L Zelter M Meininger V Derenne JP Diaphragmatic dysfunction and dyspnoea in amyotrophic lateral sclerosis Eur Respir J 2000 15 332 337 10.1034/j.1399-3003.2000.15b19.x 10706501

[B4] Arnulf I Similowski T Salachas F Garma L Mehiri S Attali V Behin-Bellhesen V Meininger V Derenne JP Sleep disorders and diaphragmatic function in patients with amyotrophic lateral sclerosis Am J Respir Crit Care Med 2000 161 849 856 10.1164/ajrccm.161.3.9805008 10712332

[B5] Bouteloup C Desport JC Clavelou P Guy N Derumeaux-Burel H Ferrier A Couratier P Hypermetabolism in ALS patients: an early and persistent phenomenon J Neurol 2009 256 1236 1242 10.1007/s00415-009-5100-z 19306035

[B6] Desport JC Preux PM Magy L Boirie Y Vallat JM Beaufrere B Couratier P Factors correlated with hypermetabolism in patients with amyotrophic lateral sclerosis Am J Clin Nutr 2001 74 328 334 11522556 10.1093/ajcn/74.3.328

[B7] Desport JC Torny F Lacoste M Preux PM Couratier P Hypermetabolism in ALS: correlations with clinical and paraclinical parameters Neurodegener Dis 2005 2 202 207 10.1159/000089626 16909026

[B8] Shimizu T Nagaoka U Nakayama Y Kawata A Kugimoto C Kuroiwa Y Kawai M Shimohata T Nishizawa M Mihara B Reduction rate of body mass index predicts prognosis for survival in amyotrophic lateral sclerosis: a multicenter study in Japan Amyotroph Lateral Scler 2012 13 363 366 10.3109/17482968.2012.678366 22632442

[B9] Clavelou P Blanquet M Peyrol F Ouchchane L Gerbaud L Rates of progression of weight and forced vital capacity as relevant measurement to adapt amyotrophic lateral sclerosis management for patient Result of a French multicentre cohort survey J Neurol Sci 2013 331 126 131 10.1016/j.jns.2013.06.002 23809193

[B10] Korner S Hendricks M Kollewe K Zapf A Dengler R Silani V Petri S Weight loss, dysphagia and supplement intake in patients with amyotrophic lateral sclerosis (ALS): impact on quality of life and therapeutic options BMC Neurol 2013 13 84 10.1186/1471-2377-13-84 23848967 PMC3717067

[B11] Attali V Mehiri S Straus C Salachas F Arnulf I Meininger V Derenne JP Similowski T Influence of neck muscles on mouth pressure response to cervical magnetic stimulation Am J Respir Crit Care Med 1997 156 509 514 10.1164/ajrccm.156.2.9612053 9279232

[B12] Brooks BR Miller RG Swash M Munsat TL El Escorial revisited: revised criteria for the diagnosis of amyotrophic lateral sclerosis Amyotroph Lateral Scler Other Motor Neuron Disord 2000 1 293 299 10.1080/146608200300079536 11464847

[B13] Andersen PM Borasio GD Dengler R Hardiman O Kollewe K Leigh PN Pradat PF Silani V Tomik B EFNS task force on management of amyotrophic lateral sclerosis: guidelines for diagnosing and clinical care of patients and relatives Eur J Neurol 2005 12 921 938 10.1111/j.1468-1331.2005.01351.x 16324086

[B14] Gonzalez-Bermejo J Morelot-Panzini C Arnol N Meininger V Kraoua S Salachas F Similowski T Prognostic value of efficiently correcting nocturnal desaturations after one month of non-invasive ventilation in amyotrophic lateral sclerosis: a retrospective monocentre observational cohort study Amyotroph Lateral Scler Frontotemporal Degener 2013 14 373 379 10.3109/21678421.2013.776086 23527531

[B15] Gonzalez-Bermejo J Perrin C Janssens JP Pepin JL Mroue G Leger P Langevin B Rouault S Rabec C Rodenstein D Proposal for a systematic analysis of polygraphy or polysomnography for identifying and scoring abnormal events occurring during non-invasive ventilation Thorax 2012 67 546 552 10.1136/thx.2010.142653 20971982

[B16] Harris J Benedict F A biometric study of basal metabolism in man Proc Natl Acad Sci 1918 4 370 373 10.1073/pnas.4.12.370 16576330 PMC1091498

[B17] Sundstrom M Tjader I Rooyackers O Wernerman J Indirect calorimetry in mechanically ventilated patients. A systematic comparison of three instruments Clin Nutr 2013 32 118 121 10.1016/j.clnu.2012.06.004 22763268

[B18] Cournand A Richards DW Jr Bader RA Bader ME Fishman AP The oxygen cost of breathing Trans Assoc Am Phys 1954 67 162 173 13216825

[B19] Silver JR The oxygen cost of breathing in tetraplegic patients Paraplegia 1963 17 204 214 14113295 10.1038/sc.1963.18

[B20] Bartlett RG Jr Brubach HF Specht H Oxygen cost of breathing J Appl Physiol 1958 12 413 424 13525304 10.1152/jappl.1958.12.3.413

[B21] Campbell EJ Westlake EK Cherniack RM Simple methods of estimating oxygen consumption and efficiency of the muscles of breathing J Appl Physiol 1957 11 303 308 13475181 10.1152/jappl.1957.11.2.303

[B22] Dodd DS Yarom J Loring SH Engel LA O2 cost of inspiratory and expiratory resistive breathing in humans J Appl Physiol 1988 65 2518 2523 3063710 10.1152/jappl.1988.65.6.2518

[B23] Margaria R Milic-Emili G Petit JM Cavagna G Mechanical work of breathing during muscular exercise J Appl Physiol 1960 15 354 358 14421029 10.1152/jappl.1960.15.3.354

[B24] Manning H McCool FD Scharf SM Garshick E Brown R Oxygen cost of resistive-loaded breathing in quadriplegia J Appl Physiol 1992 73 825 831 1400044 10.1152/jappl.1992.73.3.825

[B25] Liljestrand G Untersechungen uber die atmungsarbeit Scan Arch Physiol 1918 35 199 293 10.1111/j.1748-1716.1918.tb00716.x

[B26] Thung N Herzog P Christlieb II Thompson WM Jr Dammann JF Jr The cost of respiratory effort in postoperative cardiac patients Circulation 1963 28 552 559 10.1161/01.CIR.28.4.552 14068765

[B27] Wilson RS Sullivan SF Malm JR Bowman FO Jr The oxygen cost of breathing following anesthesia and cardiac surgery Anesthesiology 1973 39 387 393 10.1097/00000542-197310000-00008 4758346

[B28] Hugli O Schutz Y Fitting JW The cost of breathing in stable chronic obstructive pulmonary disease Clin Sci (Lond) 1995 89 625 632 8549081 10.1042/cs0890625

[B29] Gonzalez-Bermejo J Morelot-Panzini C Georges M Demoule A Similowski T Can diaphragm pacing improve gas exchange? Insights from quadriplegic patients Eur Respir J 2014 43 303 306 10.1183/09031936.00127713 24036240

[B30] Barle H Soderberg P Haegerstrand C Markstrom A Bi-level positive airway pressure ventilation reduces the oxygen cost of breathing in long-standing post-polio patients on invasive home mechanical ventilation Acta Anaesthesiol Scand 2005 49 197 202 10.1111/j.1399-6576.2004.00566.x 15715621

[B31] Sherman MS Pillai A Jackson A Heiman-Patterson T Standard equations are not accurate in assessing resting energy expenditure in patients with amyotrophic lateral sclerosis JPEN J Parenter Enteral Nutr 2004 28 442 446 10.1177/0148607104028006442 15568293

[B32] Ellis AC Rosenfeld J Which equation best predicts energy expenditure in amyotrophic lateral sclerosis? J Am Diet Assoc 2011 111 1680 1687 10.1016/j.jada.2011.08.002 22027050

[B33] Weijs PJ Hypermetabolism, is it real? The example of amyotrophic lateral sclerosis J Am Diet Assoc 2011 111 1670 1673 10.1016/j.jada.2011.08.011 22027048

[B34] Kasarskis EJ Berryman S Vanderleest JG Schneider AR McClain CJ Nutritional status of patients with amyotrophic lateral sclerosis: relation to the proximity of death Am J Clin Nutr 1996 63 130 137 8604660 10.1093/ajcn/63.1.130

[B35] Vaisman N Lusaus M Nefussy B Niv E Comaneshter D Hallack R Drory VE Do patients with amyotrophic lateral sclerosis (ALS) have increased energy needs? J Neurol Sci 2009 279 26 29 10.1016/j.jns.2008.12.027 19185883

[B36] Hoit JD Lansing RW Dean K Yarkosky M Lederle A Nature and evaluation of dyspnea in speaking and swallowing Semin Speech Lang 2011 32 5 20 10.1055/s-0031-1271971 21491355

[B37] Schols A Mostert R Cobben N Soeters P Wouters E Transcutaneous oxygen saturation and carbon dioxide tension during meals in patients with chronic obstructive pulmonary disease Chest 1991 100 1287 1292 10.1378/chest.100.5.1287 1935283

[B38] Wolkove N Fu LY Purohit A Colacone A Kreisman H Meal-induced oxygen desaturation and dyspnea in chronic obstructive pulmonary disease Can Respir J 1998 5 361 365 9832603 10.1155/1998/347020

[B39] Siirala W Olkkola KT Noponen T Vuori A Aantaa R Predictive equations over-estimate the resting energy expenditure in amyotrophic lateral sclerosis patients who are dependent on invasive ventilation support Nutr Metab (Lond) 2010 7 70 10.1186/1743-7075-7-70 20796286 PMC2939652

[B40] Gonzalez-Bermejo J Lofaso F Falaize L Lejaille M Raphael JC Similowski T Melchior JC Resting energy expenditure in Duchenne patients using home mechanical ventilation Eur Respir J 2005 25 682 687 10.1183/09031936.05.00031304 15802343

[B41] Lechtzin N Scott Y Busse AM Clawson LL Kimball R Wiener CM Early use of non-invasive ventilation prolongs survival in subjects with ALS Amyotroph Lateral Scler 2007 8 185 188 10.1080/17482960701262392 17538782

[B42] Leonardis L Dolenc Groselj L Vidmar G Factors related to respiration influencing survival and respiratory function in patients with amyotrophic lateral sclerosis: a retrospective study Eur J Neurol 2012 19 1518 1524 10.1111/j.1468-1331.2012.03754.x 22594630

